# Contrast discrimination: Second responses reveal the relationship between the mean and variance of visual signals

**DOI:** 10.1016/j.visres.2007.09.006

**Published:** 2007-12

**Authors:** Joshua A. Solomon

**Affiliations:** Department of Optometry and Visual Science, City University, London EC1V 0HB, UK

**Keywords:** Psychophysics, Contrast, Detection, Discrimination, Threshold, Uncertainty, Noise

## Abstract

To explain the relationship between first- and second-response accuracies in a detection experiment, Swets, Tanner, and Birdsall [Swets, J., Tanner, W. P., Jr., & Birdsall, T. G. (1961). Decision processes in perception. *Psychological Review, 68*, 301–340] proposed that the variance of visual signals increased with their means. However, both a low threshold and intrinsic uncertainty produce similar relationships. I measured the relationship between first- and second-response accuracies for suprathreshold contrast discrimination, which is thought to be unaffected by sensory thresholds and intrinsic uncertainty. The results are consistent with a slowly increasing variance.

## 1 Introduction

First applied to psychophysical data by Tanner and Swets (1954), signal-detection theory (SDT) posits that all stimuli elicit some sensation. However, due to noise, sensations experienced in the absence of stimulus can sometimes be more intense than sensations actually elicited by a stimulus. Crucial evidence for these faint hallucinations comes from Swets, Tanner, and Birdsall’s (1961) two-response, four-alternative forced-choice (2R4AFC) detection experiment, in which observers reported both their first and second choices for the temporal position of a visual target.^1^ SDT predicts that second-guessing should be better than chance, and this is what Swets et al. found.

### 1.1 Signal-detection theory

According to SDT (Green & Swets, 1966), each stimulus *X*, gives rise to a Gaussian probability-density function (PDF)

(1) $$f_{X}(x)=f(x\text{;}\mu_{X}\text{,}\sigma_{X})=\frac{1}{\sqrt{2\pi }\sigma_{X}}\exp\left[-\frac{{\left(x-\mu_{X}\right)}^{2}}{2\sigma_{X}^{2}}\right]\text{,}$$

of sensory intensity *x*. In its simplest form, all PDF’s have the same standard deviation, i.e., *σ*_*X*_ = *σ*,  ∀*X*.

### 1.2 Increasing variance

Simple SDT proved incapable of explaining Swets et al.’s (1961) 2R4AFC detection experiment. Instead, they proposed that sensation variance increased with sensation mean:

(2) $$\sigma_{X}=r\mu_{X}+1\text{,}\;\mu_{X}⩾0\text{.}$$

Increases in the “sigma-to-mean ratio”^2^ *r* = d*σ*_*X*_/d*μ*_*X*_, produce decreases in both first- and second-response accuracies, but the second-response accuracies to decrease faster. Swets et al. tried several values for this ratio and obtained as good fit to their data when *r* = 0.25.

### 1.3 Intrinsic uncertainty

Elsewhere (Solomon, 2007), Swets et al.’s (1961) 2R4AFC data have been successfully fit with an alternative model, incorporating intrinsic uncertainty. Intrinsic-uncertainty models posit that perceived intensity depends on the maximum activity in several independent sensory mechanisms, only one of which is actually sensitive to the stimulus. Given a sufficient number *M*, of these mechanisms, the variance of their maximum activity will decrease as the intensity of the stimulus decreases (see Pelli, 1985, for a graphical demonstration of this). Thus, to some degree, intrinsic uncertainty mimics Swets et al.’s proposal of increasing variance. With suprathreshold stimuli, the maximum activity always occurs in the appropriately tuned mechanism, and the others have no influence on perception.

### 1.4 Low-threshold theory

At odds with SDT is the idea of a sensory threshold, which weak stimuli must exceed to be detected. In a detection task, stimuli that do not exceed the threshold can be selected only when no other stimulus exceeds it, and the observer is forced to make a choice. Swets et al. (1961) developed this idea into a “low-threshold” hybrid of signal-detection and threshold theories. Unlike other models with this name, Swets et al. claimed theirs could fit the 2R4AFC results. Elsewhere (Solomon, 2007), I have corroborated this claim, and shown that the fit is not quite as good as those produced by models including either intrinsic uncertainty or increasing variance.

### 1.5 This study

Neither intrinsic uncertainty (Pelli, 1985; Tanner, 1961) nor Swets et al.’s (1961) low-threshold theory requires sensation variance to increase with sensation mean. These theories are somewhat special because they are thought to affect the visibility of only very faint stimuli. Increasing variance, on the other hand, has implications for suprathreshold contrast discrimination. For this reason, I decided to conduct a 2R4AFC contrast-discrimination experiment. The goal was to obtain an estimate of the sigma-to-mean ratio, which would not be contaminated by intrinsic uncertainty or a low threshold.

## 2 Methods

There were five observers: the author (JAS), another psychophysicist who understood the purposes of the experiment (MJM), two experienced psychophysical observers who were naïve to the purposes of this experiment (FV and MT) and one further observer who had no previous laboratory experience (NN). As described below, NN produced a very high proportion of “finger errors.” This suggested to us a general unreliability, and no further analyses were performed on his data.

**Fig. 1:**
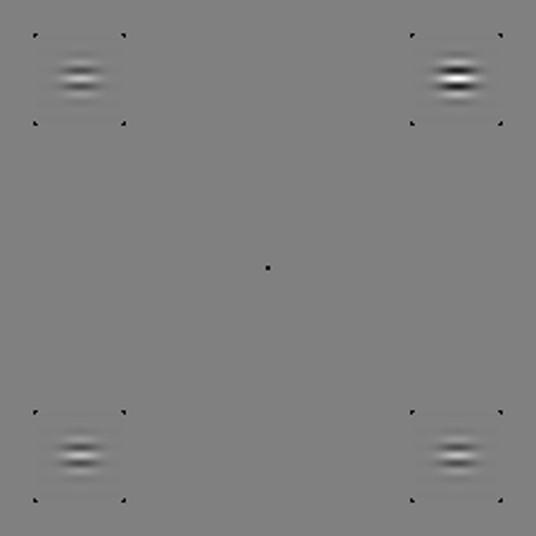
Example stimulus. One Gabor has more contrast than the others. When those others have sufficient “pedestal” contrast for essentially perfect detection, neither intrinsic uncertainty nor a sensory threshold can contaminate an observer’s decision as to which of the four is most intense. For JAS and MJM, all of the black spots disappeared during each 0.18-s stimulus exposure. For the other observers, only the central fixation spot disappeared.

The Psychophysica (Watson & Solomon, 1997a) software used in these experiments is available at http://vision.arc.nasa.gov/mathematica/psychophysica.html. The 23.5-cd/m^2^ display (a Sony GDM F-520 CRT) was viewed in a dark room from 1.15 m. Luminances of vertically adjacent pixels were effectively independent, and could obtain any value between 1.06 and 46 cd/m^2^. Stimuli were horizontal, cosine-phase Gabor patterns whose wavelength and spatial spread were *λ* = 0.25° and *σ* = 0.18°, respectively. Stimuli were flashed simultaneously, in four positions, each marked by four dark spots. The centers of these positions formed a 5.6° × 5.6° square centered on fixation (see Fig. 1).

On each trial, three stimuli appeared with a pedestal contrast, which varied between blocks of 90 trials each. The contrast of the fourth stimulus was somewhat greater. After each 0.18-s stimulus exposure, observers gave two responses. The first response indicated which of the four positions the observer thought most likely to have contained the high-contrast target. The second responses from JAS and MJM indicated their second choices for the target position. Following their second responses, JAS and MJM received auditory feedback indicating which—if either—of their responses was correct.

The naïve observers were not told that three of the four stimuli would have the same contrast. They were instructed merely to indicate their choices for the positions containing the two highest contrasts, in order. This encouraged them to fully consider their second responses, even when they felt confident about their first. The naïve observers received no feedback.

**Fig. 2:**
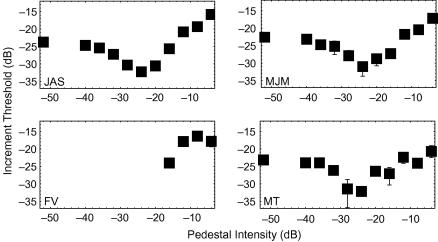
Threshold-vs.-contrast functions for first responses in four observers. Error bars contain 95% confidence intervals.

Although I was primarily concerned with suprathreshold contrast discrimination, I was also eager to replicate Swets et al.’s (1961) findings at detection threshold. Since I was therefore committed to measuring both the left and right ends of the threshold-vs.-contrast function (Nachmias & Sainsbury, 1974; Fig. 2), I decided to devote a few extra trials to get the middle as well. (Note: due to limited availability, FV performed only the critical conditions, i.e., those with supra-threshold pedestals.)

Table 1 shows the number of trials each observer performed with each pedestal contrast. In it, and in the discussion below, I use the conventional decibel scale of contrast energy: if *m* is the maximum available contrast, then an *x* dB stimulus is one that has a contrast of *m*10^*x*/20^.

Prior to each trial, the quest procedure (Watson & Pelli, 1983) estimated the performance threshold *c*_t_, i.e., the contrast increment required for 62%-accurate first responses. This is halfway between chance performance (25%) and a hypothetical ceiling of 99%.

As a means of more accurately estimating the ceiling, or equivalently, the frequency of finger errors, a −10 dB increment was used on one-ninth of the trials. For JAS, the target was given an increment of either *c*_t_ − 2 dB or *c*_t_ + 2 dB, with equal probability, on the remaining trials. For the other observers, target increments were either *c*_t_ − 2 dB or *c*_t_ . This modification allowed better sampling of their psychometric functions. Finally, to further encourage the naïve observers to fully consider their second responses, one of the three alternatives to each of the “obvious” (−10 dB) targets was fixed at −16 dB.

## 3 Results

### 3.1 Psychometric functions

For each observer and each pedestal, first-response accuracies were maximum-likelihood^3^ fit with a modified Gaussian distribution.

(3) $$\Psi_{1}(c)=0.25+(0.75-\delta )\int_{-\infty }^{c}f(u\text{;}c_{\text{t}}\text{,}\sigma )du\text{.}$$

In the preceding expression, *c* is the increment (in dB), *Ψ*_1_ is the probability of a correct first response and *f* is the PDF defined in Eq. (1). Threshold *c*_t_, and *σ* were free parameters, but the frequency of finger errors *δ*, was not allowed to vary with pedestal contrast. These psychometric fits were obtained for purely descriptive purposes. Unlike some of the fits described below, these were not driven by any particular model of performance. Best-fitting values for *δ* were 0.006, 0.002, 0.048, 0.018 and 0.095 for JAS, MJM, FV, MT and NN, respectively. When debriefed, NN reported a tendency to respond before the end of a trial.

### 3.2 Threshold-vs.-contrast functions (first response only)

Fig. 2 shows how threshold varies with pedestal contrast. JAS’s and MJM’s thresholds were similar, and formed the classic “dipper” shaped function. MT did not suffer as much masking. That is, his thresholds with high-contrast pedestals were lower than the JAS’s and MJM’s. However, his detection threshold—obtained with pedestals having zero contrast (or −∞ dB)—was similar: between −24 and −23 dB. FV’s thresholds with high-contrast pedestals fall within the range spanned by the other observers’, thus we can be reasonably confident that these pedestals exceeded her detection threshold, as they did for the other observers.

### 3.3 Second-vs.-first-response-accuracy functions: Detection

**Fig. 3:**
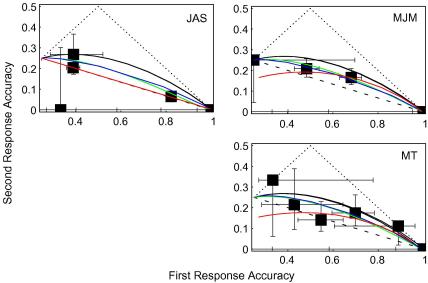
Two-response, four-alternative-forced-choice (2R4AFC), detection results for three observers. Each box represents a unique intensity. Error bars contain 95% confidence intervals. The solid black curves show simple signal-detection theory (SDT). Dashed lines show high-threshold theory. Dotted lines are mathematical and theoretical upper bounds for second-response accuracies. The green, blue and red curves show, respectively, the maximum-likelihood fits of SDT modified with increasing noise, intrinsic uncertainty and a low threshold.

Fig. 3 shows how first- and second-response accuracies co-varied when, as in Swets et al.’s (1961) experiment, there was no pedestal. Appendix A contains a full description of the raw data. Several features of Fig. 3 deserve a detailed description.

The axes differ from those used by Swets et al. (1961). For the horizontal axes, instead of signal strength, which may not be a linear function of contrast, I prefer first-response accuracy *Ψ*_1_. For the vertical axes, Swets et al. used second-response accuracy, divided by the proportion of first-response errors. However, both first- and second-response accuracies are subject to measurement error. When one uncertain statistic is divided by another, the confidence intervals for the quotient are necessarily very large. To avoid this problem, I get rid of the denominator and plot simply second-response accuracy *Ψ*_2_.

One argument against this way of plotting the results is that a large portion of the graph will be wasted because *Ψ*_2_ ⩽ 1 − *Ψ*_1_. A dotted line has been added to each graph to indicate this upper limit for second-response accuracy. Similarly, *Ψ*_2_ ⩽ *Ψ*_1_. Of course, given a finite number of trials, we may find that the frequency of correct second responses *P*_2_ exceeds the frequency of correct first responses *P*_1_, but only a perverse observer would have a greater *probability* of being correct in his or her second response. Therefore I have added another dotted line to each graph to indicate this other upper limit for second-response accuracy.

Each point in Fig. 3 represents data collected with a unique target contrast. Several of these points reflect only a few responses made at the beginning of the experiment, before the adaptive staircase had converged. We can thus have little confidence in the likelihood of a correct first or second response with these targets. To convey this confidence (or lack thereof), I have plotted 95% confidence intervals, both horizontally and vertically, about each point. These intervals are based on binomial probabilities, calculated from the range defined by the limits described in the preceding paragraph (see Appendix B for details).

### 3.4 Modeling finger errors

SDT can be modified to accommodate finger errors. Let *ψ*_1_ denote the first-response accuracy without errors. For those trials containing a first-response finger error, the probability of a correct first response is (1 − *ψ*_1_)/3. Thus, if the finger-error rate is *δ*, the overall probability of a correct first response is (1 − *δ*)*ψ*_1_ + *δ*(1 − *ψ*_1_)/3.

To derive the formula for second-response accuracy, it helps to understand that first-response finger-errors will be incorrect with probability 1 − [(1 − *ψ*_1_)/3] = (2 + *ψ*_1_)/3. Without loss of generality, we may assume that observers correct some proportion *ε* of first-response finger errors with their second response. Thus on these trials, the probability of a correct second response is *ψ*_1_. When first-response finger-errors are not explicitly corrected, I will assume that the second response is completely random. On these latter trials, the second response will be correct with probability [(2 + *ψ*_1_)/3]/3 = (2 + *ψ*_1_)/9. Thus, on those trials in which a first-response finger error occurred, the second-response accuracy should be

(4) $$\Psi_{2}^{\prime }=\varepsilon \psi_{1}+(1-\varepsilon )(2+\psi_{1})/9\text{.}$$

Second-response accuracy overall will be (1 − *δ*)*ψ*_2_ + *δ* [*εψ*_1_ + (1 − *ε*)(2 + *ψ*_1_)/9], where *ψ*_2_ would have been the second-response accuracy, had there been no first-response finger errors.

To estimate *ε*, trials containing an “obvious” (−10 dB) increment and an incorrect first response were examined. (Because the highest pedestals had the potential for masking even these large increments, they were excluded from this analysis.) On these trials, we may assert that *ψ*_1_ = 1, and we can then solve Eq. (4) for *ε*. Solutions were 0.70, 0, 0.58 and 0 for JAS, MJM, FV and MT, respectively.

### 3.5 Maximum-likelihood fits

**Fig. 4:**
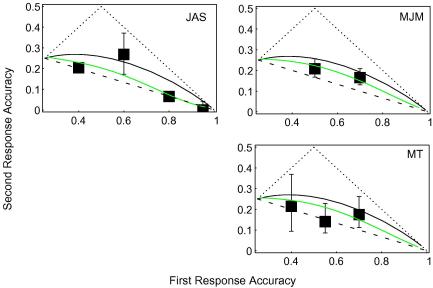
Cleaned-up 2R4AFC detection results (see text). Green curves show the best fit of SDT with increasing variance. (For interpretation of the references to colour in this figure legend, the reader is referred to the web version of this article.)

**Fig. 5:**
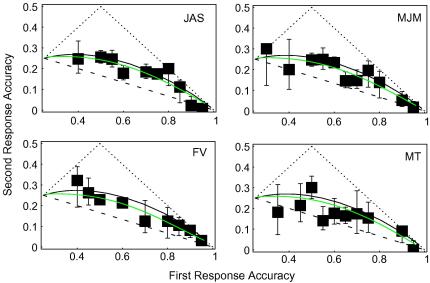
2R4AFC contrast-discrimination results. Green curves show the best fit of SDT with increasing variance. (For interpretation of the references to colour in this figure legend, the reader is referred to the web version of this article.)

**Fig. 6:**
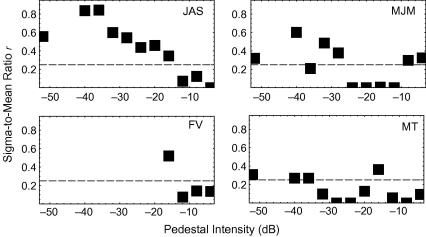
How the sigma-to-mean ratio varies with signal intensity. Dashed lines indicate Swets et al.’s (1961) estimate, based on detection data.

These values of *ε* were assumed when calculating the curves in Figs. 3–6. In Fig. 3, the solid black curves represent the prediction of simple SDT, that is, when *r* = 0 in Eq. (2). The dashed lines represent the prediction of high-threshold theory, which ascribes all errors to unlucky guesses rather than faint hallucinations. (See Swets et al., 1961 or Solomon, 2007, for derivations of these predictions.)

Maximum-likelihood fits were also obtained for three further modifications of SDT. They were: (i) increasing variance with power-law transduction, (ii) intrinsic uncertainty and (iii) low-threshold theory. Details of these three models can be found in Appendix C, and receiver-operating characteristics for all three models applied to a yes/no-detection task appear in Nachmias (1972). The fits appear as solid green, blue and red curves, respectively, in Fig. 3.

Goodness-of-fit is indicated by the generalized (log) likelihood-ratios in Table 2. Specifically, these values reflect the maximum log likelihoods, minus the conventional upper-bound on log likelihood described in Footnote 3. Note that unlike some generalized likelihood-ratios (Mood, Graybill, & Boes, 1974), these cannot be expected to follow the chi-square distribution because there are so many conditions (i.e., specific increment contrasts) containing only one or two trials (Wichmann & Hill, 2001).

Only for JAS, low-threshold theory produced, by far, the best fits of the three SDT models. In fact, the best-fitting “low” threshold for JAS was effectively a high threshold, never exceeded by zero-contrast pedestals. That is why JAS’s red curve in Fig. 3 is visually indistinguishable from the dashed black line. Since JAS’s results are consistent with a high threshold, they are inconsistent with the findings and conclusions of Swets et al. (1961).

For the other two observers, the sigma-to-mean ratios of the best-fitting increasing-variance models were between 0.26 and 0.28; similar to Swets et al.’s (1961) estimate of 0.25. Thus, these results can be considered a successful replication of Swets et al.’s. At the end of this section, I speculate on why JAS’s results differ.

### 3.6 Transducer-independent estimates of *r*

At detection threshold, as described above, the raw data were simply fit with a multiplicative-noise/power-law-transducer version of SDT. However, with suprathreshold pedestals, we cannot be certain what shape the transducer takes. Increasing-variance models (e.g., Kontsevich, Chen, & Tyler, 2002) use a simple power-law transducer, but constant-noise models (e.g., Legge & Foley’s, 1980), use a transducer that is initially expansive, then compressive, as the pedestal increases. The compressive non-linearity is required to produce masking; i.e., threshold elevation from high-contrast pedestals.

We do not really have to worry about the form of transducer, because SDT’s predictions for the relationship between first- and second-response accuracies are independent of signal transduction (Solomon, 2007; Swets et al., 1961). We merely need to quantify how these predictions change with the sigma-to-mean ratio, and find the values most consistent with the data. This examination of a transducer-independent facet of contrast-discrimination data is complementary to attempts at modeling contrast-discrimination data without putting any constraint on the form of the transducer (Katkov, Tsodyks, & Sagi, 2006a; Katkov, Tsodyks, & Sagi, 2006b; Klein, 2006).

Transducer-independent estimates of the sigma-to-mean ratio *r* were obtained by maximizing the likelihood of observing second-response accuracies *P*_2_, given the first-response accuracies *P*_1_. A complete description of this process appears in Appendix D. The best fits were 0.56 for JAS, 0.32 for MJM and 0.31 for MT. These values are illustrated in Fig. 4. All of these fits are comparable to the maximum-likelihood fits described above.

### 3.7 Binning accuracy

Fig. 4 is less cluttered than Fig. 3. It has fewer data points and no horizontal confidence intervals. Nonetheless, the same data appear in both figures. For legibility, I have decided to combine data from increments producing similar first-response accuracies, as determined by the psychometric functions described above. I have adopted the relatively arbitrary decision to use 5 dB bin-widths. There is one visible consequence of this manipulation: a rightward shift for one data point in JAS’s panel. However, without binning, the suprathreshold data presented below would be impossible to read.

I have also decided to forgo plotting data from increments producing both floor (i.e., 0.25) and ceiling (i.e., 1 − *δ*, see Eq. (3)) accuracies. Such data are worthless at discriminating between candidate models. Finally, also for the sake of legibility, I have also decided to cull data points representing fewer than 10 trials. Of course, these latter data are not completely worthless; they have not been excluded from any model fits. A complete description of the binned data appears in Appendix A.

### 3.8 Second-vs.-first-response-accuracy functions: Suprathreshold discrimination

Fig. 5 shows how first- and second-response accuracies co-varied for the four highest pedestals. (The binned data are tabulated in Appendix A.) Plotting conventions have been inherited from Fig. 4.

It should be apparent that the second responses with these suprathreshold pedestals tend to be more accurate than the second responses at detection threshold. Using the same procedure as was described for the detection data (above and in Appendix D), values for the sigma-to-mean ratio *r* were found that maximized the likelihood of observing second-response accuracies *P*_2_, given the first-response accuracies *P*_1_. The best fits were 0.11 for JAS, 0.16 for MJM, 0.21 for FV and 0.09 for MT. These values are illustrated in Fig. 5.

These values of *r* are smaller than those required to fit the detection data. FV’s relatively high value may have something to do with her relatively high finger-error rate. No detection data from FV are available for comparison.

Pedestal-by-pedestal estimates of *r* appear in Fig. 6. Most of these estimates remain near or below the value of 0.25, selected by Swets et al. (1961), when neither the effect of a low threshold nor that of intrinsic uncertainty is considered. The results from JAS are different; best-fitting values of *r* start at 0.56, and decrease to 0 as pedestal intensity increases.

JAS’s small-pedestal estimates of *r* are strangely high. As noted above, they are not incompatible with the notion that visual noise never exceeds the threshold of visibility in the absence of a stimulus. Previous attempts to replicate Swets et al.’s (1961) 2R4AFC results have also met with mixed success. Despite their chronological precedence, Kincaid and Hamilton’s (1959) results have been described as both successful and unsuccessful replications of Swets et al.’s (Green & Swets, 1966 and Blackwell, 1963, respectively). As yet, I have not been able to track down a copy of Kincaid and Hamilton’s publication. One further attempt to replicate Swets et al. was described by Eijkman and Vendrik (1963). They reported that second responses for light detection were greater than chance in just one of three observers.

An alternative interpretation for JAS’s high-threshold-like performance is that he simply ignored sensory information and selected his second responses more-or-less randomly. This explanation would be easier to swallow if all his estimates of *r* were high. However, with suprathreshold pedestals, his *r* is no higher than that of the other observers. Expectation may have caused JAS to change his strategy with pedestal intensity, but I have no definitive answer.

## 4 Discussion

The most equitable summary of these results is that they are consistent with a performance-limiting source of noise, which increases slightly with suprathreshold contrast.

Best estimates for the rate varied from 0.09 to 0.21. I wondered whether such small sigma-to-mean ratios would be sufficient to model the high contrast-discrimination thresholds obtained with high-contrast pedestals, or whether a saturating transducer function for stimulus contrast would also be necessary. Previous attempts (Kontsevich et al., 2002; Solomon, 2007) to fit contrast-discrimination data without compressive transduction have not focused on the minimum necessary sigma-to-mean ratio, but that ratio can be inferred from the published parameter values.

### 4.1 Fitting contrast discrimination

In those previous attempts, the standard deviation of visual signals was allowed to increase as a decelerating power function of the mean. Specifically,

(5) $$\sigma =r\mu^{q}+\sigma_{0}\text{,}\;0<q<1\text{.}$$

(Kontsevich et al. considered only suprathreshold contrast and thus could set *σ*_0_ = 0; Solomon used *σ*_0_ = 1.) Therefore, the sigma-to-mean ratios decreased as the means increased. From the best-fitting parameter values, I have used a contrast of 100% to infer the minimum sigma-to-mean ratios required to explain contrast discrimination without compressive transduction. The smallest of these ratios was 0.13 (obtained using the parameter values fit to observer SV with “sustained” stimuli in Kontsevich et al., 2002). Thus it seems that the sigma-to-mean ratios estimated in the present study may in fact be able to produce appreciable masking.

Four different models were maximum-likelihood fit to all of JAS’s, MJM’s and MT’s 2R4AFC data. Details of all four models appear in Solomon (2007). One of these models was a four-parameter, non-linear transducer model with constant Gaussian noise (i.e., where *r* = 0). This transducer is initially expansive, then compressive, as the input increases. Foley (1994) obtained good fits to 2AFC contrast-discrimination thresholds with this model, but we already know this model will over-estimate second-response accuracies, particularly in a (zero-pedestal) detection experiment (Solomon, 2007; Swets et al., 1961).

In the other three models that were fit to JAS’s, MJM’s and MT’s data, the variance of visual signals was allowed to increase with the mean. Two of these models have already proven capable of producing acceptable fits to some of Foley’s (1994) 2AFC thresholds (see Fig. 13 of Solomon, 2007). In one of these, a power-law transducer is responsible for the ‘dip’ in the contrast-discrimination function. In the other, intrinsic uncertainty produces the dip. The remaining increasing-variance model explored in this paper uses a low threshold to produce the dip.

Fit details appear in Table 3. Initially, four parameters were allowed to vary freely in each fit. For JAS and MJM the best-fitting model was the one with increasing variance model and intrinsic uncertainty. In general, the fits improved as uncertainty *M*, increased. For JAS and MJM, the fits of the increasing-variance/intrinsic-uncertainty model remained superior when *M* was fixed at a value of 10,000. When the relationship between signal mean and standard deviation was forced to be linear (i.e., *q* = 1 in Eq. (5)), the fits of the increasing-variance/intrinsic-uncertainty model remained superior. They even remained superior when *M* was fixed at a value of 1000. Thus, all of JAS and MJM’s data can be satisfyingly summarized by a relatively simple model, combining increasing variance with intrinsic uncertainty. Within the context of this model the best-fitting values of *r* (regardless of constraints on *M* or *q*) were 0.16 for JAS and 0.14 for MJM. These values are nearly identical to the transducer-independent estimates (0.14 for JAS and 0.16 for MJM), described above.

When MT’s data were fit with a 3-parameter increasing-variance/intrinsic uncertainty model, the best-fitting values for the sigma-to-mean ratio and intrinsic uncertainty were *r* = 0.11 and *M* = 440, respectively. However, his data are best-fit by the constant-variance, 4-parameter, nonlinear-transducer model (Foley, 1994). Thus, in two out of three cases, the contrast-discrimination data can be satisfyingly summarized by a 3-parameter model of intrinsic uncertainty and increasing variance. Compressive transduction is not required.

### 4.2 Sensory thresholds for contrast discrimination

Although “sensory threshold” means different things to different people (Swets et al., 1961), it is usually understood to be some sort of barrier weak stimuli must overcome to be perceived (Swets, 1961). However, it is no less valid to apply the concept to the task of contrast discrimination, even with large pedestals. That is, forced-choice errors may occur simply because all the alternatives appear identical and the observer simply guesses incorrectly.

Inspection of Fig. 5 should be sufficient to rule out any “high” sensory threshold for contrast discrimination. The data shown there are even less similar to the (dashed) high-threshold prediction than Swets et al.’s (1961) detection data (not shown). However, some proportion of correct second responses may indeed have been just lucky guesses, which is to say my data cannot rule out a “low threshold” for “suprathreshold” contrast discrimination.

### 4.3 Other models

The logic of this study hinges on the assumption that unmasked, suprathreshold contrast discrimination can be modeled with a single sensory mechanism. Although this assumption is popular, it is not completely uncontroversial. Before I describe other possible models, I should first stress the importance of the word “unmasked.” In a highly influential paper, Foley (1994) argued that the mechanism responsible for contrast discrimination was not immune to the activity in differently tuned mechanisms. By manipulating the spatial phase, orientation and temporal frequency of masking stimuli, Foley and Boynton (1994) were able to probe the interactions between mechanisms responsible for contrast discrimination. However, when no masks are present, most models (including all of Foley’s) consider contrast discrimination to be mediated by a single mechanism or channel; the one best tuned to the target.

There are three notable exceptions. Teo and Heeger (1994) and Yu, Klein, and Levi (2004) have developed models with greater physiological plausibility, in which individual mechanisms have very limited dynamic ranges. Elsewhere (Watson & Solomon, 1997b) I have argued this type of model is well approximated by the more popular, single-mechanism model for contrast discrimination. The two types of model can be considered equivalent when performance-limiting noise is added after the outputs of multiple mechanisms are combined.

The third exception was recently proposed by Henning and Wichmann (2007) to account for their finding that the low-contrast “dip” of threshold-vs.-contrast functions (e.g., Fig. 2) disappears in the presence of a notched-noise mask. This result suggests the dip is due to off-frequency looking. That interpretation may be correct, but I am obligated to note their results are also consistent with intrinsic uncertainty, which would attribute a less-pronounced dip to uncertainty reduction. Indeed, Blackwell (1998) argued that noise, both within the detector’s pass band and outside it, could reduce intrinsic uncertainty and facilitate detection. She also provided psychophysical evidence for this facilitation.

## Figures and Tables

**Table A1:** Raw detection data

Observer	Increment (dB)	Number of trials	First-response accuracy (*P*_1_)	Second-response accuracy (*P*_2_)
JAS	−34	1	0	0
JAS	−28	3	0.33	0
JAS	−26	751	0.38	0.20
JAS	−24	41	0.39	0.27
JAS	−22	755	0.82	0.06
JAS	−20	45	1	0
JAS	−18	2	1	0
JAS	−10	202	1	0
MJM	−44	1	0	0
MJM	−38	1	1	0
MJM	−36	1	0	1
MJM	−28	1	0	0
MJM	−26	4	0.25	0.25
MJM	−24	221	0.48	0.21
MJM	−22	248	0.68	0.17
MJM	−20	2	1	0
MJM	−18	1	1	0
MJM	−10	60	1	0
MT	−44	1	0	0
MT	−38	1	1	0
MT	−32	1	0	0
MT	−30	1	0	1
MT	−28	3	0.33	0.33
MT	−26	14	0.43	0.21
MT	−24	64	0.55	0.14
MT	−22	63	0.70	0.17
MT	−20	9	0.89	0.11
MT	−18	1	1	0
MT	−10	22	1	0

**Table A2:** Binned detection data

Observer	Number of trials	First-response accuracy (*Ψ*_1_ ± 0.025)	Second-response accuracy (*P*_2_)
JAS	3	0.30	0
JAS	751	0.40	0.20
JAS	41	0.60	0.27
JAS	755	0.80	0.06
JAS	45	0.95	0
MJM	4	0.35	0.25
MJM	221	0.50	0.21
MJM	248	0.70	0.17
MJM	2	0.85	0
MJM	1	0.95	0
MT	3	0.30	0.33
MT	14	0.40	0.21
MT	14	0.40	0.21
MT	64	0.55	0.14
MT	63	0.70	0.17
MT	9	0.85	0.11
MT	1	0.95	0

**Table A3:** Binned contrast-discrimination data

Observer	Number of trials	First-response accuracy (*Ψ*_1_ ± 0.025)	Second-response accuracy (*P*_2_)
JAS	6	0.30	0.50
JAS	2	0.35	0
JAS	81	0.40	0.45
JAS	7	0.45	0.14
JAS	559	0.50	0.26
JAS	309	0.55	0.25
JAS	327	0.60	0.18
JAS	9	0.65	0.11
JAS	337	0.70	0.18
JAS	780	0.75	0.17
JAS	79	0.80	0.22
JAS	18	0.85	0.11
JAS	91	0.90	0.02
JAS	266	0.95	0.01
MJM	16	0.30	0.31
MJM	7	0.35	0.14
MJM	25	0.40	0.20
MJM	8	0.45	0.38
MJM	499	0.50	0.25
MJM	189	0.55	0.25
MJM	699	0.60	0.23
MJM	227	0.65	0.15
MJM	170	0.70	0.15
MJM	46	0.75	0.20
MJM	29	0.80	0.14
MJM	7	0.85	0
MJM	58	0.90	0.05
MJM	53	0.95	0.02
FV	4	0.30	0.50
FV	6	0.35	0.17
FV	28	0.40	0.32
FV	122	0.45	0.26
FV	910	0.50	0.23
FV	769	0.60	0.21
FV	48	0.70	0.12
FV	5	0.75	0
FV	16	0.80	0.12
FV	57	0.85	0.11
FV	111	0.90	0.08
FV	63	0.95	0.03
MT	6	0.30	0.60
MT	18	0.35	0.18
MT	52	0.45	0.21
MT	191	0.50	0.30
MT	105	0.55	0.14
MT	102	0.60	0.18
MT	234	0.65	0.16
MT	16	0.70	0.17
MT	47	0.75	0.15
MT	8	0.85	0
MT	28	0.90	0.09
MT	27	0.95	0

Only data from the four highest pedestals appear in this table.

**Table D1:** Generalized (natural) log-likelihood-ratios for models fit to second-response accuracy

Task	Model	JAS	MJM	FV	MT
Detection (Fig. 4)	Constant noise (0)	−26.1	−3.0		−2.8
	Increasing variance (1)	−5.5	−1.1		−2.1
					
Suprathreshold discrimination (Fig. 5)	Constant noise (0)	−16.6	−14.6	−14.5	−10.0
	Increasing variance (1)	−15.2	−12.7	−11.6	−9.8

The constant noise model has no free parameters. Second-response accuracy is completely determined by first-response accuracy. The increasing variance model has one free parameter: the sigma-to-mean ratio *r*.

**Table 1:** Number of trials each observer performed with each pedestal

Pedestal (dB)	JAS	MJM	FV	MT
−∞	1800	540	0	180
−40	360	180	0	180
−36	360	180	0	180
−32	360	180	0	180
−28	360	180	0	180
−24	360	180	0	180
−20	360	180	0	180
−16	720	540	540	180
−12	720	540	540	180
−8	720	540	540	180
−4	720	540	540	180

**Table 2:** Generalized (natural) log-likelihood-ratios for models fit to the (zero-pedestal) detection data

	JAS	MJM	MT
Power-function transducer with increasing variance (3)	−14.6	−5.4	−7.9
Intrinsic uncertainty (2)	−18.6	−5.3	−6.9
Low threshold (2)	−7.4	−9.7	−7.5

The number of free parameters appears in parentheses.

**Table 3:** Generalized (natural) log-likelihood-ratios for models fit to the entire dataset

Model	Parameters	JAS	MJM	MT
Foley (1994)	*a*	−207	−97	−96
	*Z*			
	*q*			
	*p*			
				
Increasing	*a*	−248	−114	−115
Variance	*r*			
Power-law	*q*			
Transduction	*p*			
				
Increasing	*a*	−176	−107	−126
Variance	*r*			
Low	*q*			
Threshold	*c*			
				
Increasing	*a*	−167	−93	−105
Variance, Intrinsic	*r*			
Uncertainty	*q* = 1			
	*M* ⩽ 10,000			
				
Increasing	*a*	−184	−94	−105
Variance, Intrinsic	*r*			
Uncertainty	*q* = 1			
	*M* = 1000			

The parameter notation is that used by Solomon (2007; see also Appendix C). The only fixed parameter values were those indicated by equations in the second column.

## Appendix A

Tables A1–A3.

## Appendix B

For each increment and pedestal contrast, responses may be considered to adhere to the Bernoulli distribution, where the probability of being correct is given by the parameter *Ψ*. Let *NP* denote the number of correct responses in *N* trials. If we have no *a priori* reason to constrain the value of *Ψ*, then the probability that *Ψ* ⩽ *p* is given by the binomial distribution *G*(*NP*; *N*, *p*), for any 0 ⩽ *p* ⩽ 1. However, if we know that 0 ⩽ *p*_min_ ⩽ *Ψ* ⩽ *p*_max_ ⩽ 1, the probability that *Ψ* ⩽ *p* becomes

(6) $$h(p)=\int_{p_{\min}}^{p}g(\mathrm{NP}\text{;}N\text{,}v)/\int_{p_{\min}}^{p_{\max}}g(\mathrm{NP}\text{;}N\text{,}u)dudv)\text{,}$$

where *g* = *G*′ is the binomial PDF. Ninety-five percent confidence intervals for estimates of *Ψ* can be obtained by evaluating the inverse functions *h*^−1^(0.025) and *h*^−1^(0.975).

## Appendix C

Formulae appearing in this appendix may be used to construct all of the models described above. Derivations of these formulae appear in Solomon (2007), but finger errors were not considered in that paper. Here, I reserve (capital) *Ψ* for probability correct and (lower case) *ψ* for probability correct *in the absence of finger errors*. Formulae for converting *ψ* to *Ψ* appear in Section 3.4.

In general, first and second-response accuracies are given by

(7) $$\psi_{1}=\int_{-\infty }^{\infty }{\left[F_{N}(x)\right]}^{3}f_{S}(x)dx$$

and

(8) $$\psi_{2}=\frac{3\int_{-\infty }^{\infty }{\left[F_{N}(x)\right]}^{2}f_{S}(x)\left[1-F_{N}(x)\right]dx}{1-\int_{-\infty }^{\infty }{\left[F_{N}(x)\right]}^{3}f_{S}(x)dx}\text{.}$$

In the preceding expressions, *F*_*N*_(*x*) is the cumulative distribution (CDF) of sensations arising from each pedestal and *f*_*S*_(*x*) is the PDF of sensations arising from the target + pedestal. To obtain the latter, its CDF must be differentiated:

(9) $$f_{S}(x)=F_{S}^{\prime }(x)\text{.}$$

CDF’s for the pedestal (where *X* = *N*) and target (i.e., pedestal plus increment; *X* = *S*) are given by the formula:

(10) $$F_{X}(x)=\left\{\begin{array}{cc} {\left[F(x\text{;}0\text{,}1)\right]}^{M-1}F(x\text{;}\mu_{X}\text{,}r\mu_{X}^{q}+1) & x⩾c\\ {\left[F(c\text{;}0\text{,}1)\right]}^{M-1}F(c\text{;}\mu_{X}\text{,}r\mu_{X}^{q}+1) & 0⩽x<c\text{,}\\ 0 & \text{otherwise} \end{array}\right)$$

where *M* is the number of (independent) sensory mechanisms upon which perceived intensity depends (i.e., the degree of intrinsic uncertainty), $F(x\text{;}\mu \text{,}\sigma )=\int_{-\infty }^{x}f(u\text{;}\mu \text{,}\sigma )du$ is the cumulative Guassian distribution (see Eq. (1)), *μ*_*X*_ and $r\mu_{X}^{q}+1$ are the mean and standard deviation (see Eq. (5)) of activity in the single mechanism that is actually sensitive to each Gabor pattern and *c* is the sensory threshold. When there is no sensory threshold, *c* = −∞; when there is no intrinsic uncertainty, *M* = 1.

The expression for power-law transduction is

(11a) $$\mu_{X}={\mathrm{at}}_{X}^{p}\text{,}$$

where *t*_*X*_ denotes either pedestal or target contrast, depending on whether *X* = *N* or *X* = *S*. The parameters *a* and *p* are allowed to vary. When *p* = 1, transduction is linear. The equation for Foley’s (1994) four-parameter transducer is

(11b) $$\mu_{X}=\frac{{\mathrm{at}}^{p}}{Z+t_{X}^{q}}\text{.}$$

The parameters *a*, *Z*, *p* and *q*, are all allowed to vary. The *q* appearing Eq. (11b) is different from that appearing in Eqs. (5) and (10), however no confusion should arise because I do not combine Foley’s transducer with increasing variance, i.e., when Eq. (11b) is used, the other *q* gets set to 0.

## Appendix D

According to signal-detection theory, contrast discrimination depends on the expected difference between visual signals elicited by the high- and low-contrast patterns. In other words, it is their relative intensities that matter, not their absolute intensities. Therefore, with no loss of generality, I may declare that a pedestal can elicit sensations having a mean intensity *μ*_*N*_ = 0. It follows that the pedestal + increment must then elicit an average signal *μ*_*S*_ > 0.

Brent’s method was used to find this average signal *μ*_*S*_, which was required to produce a criterion first-response accuracy *Ψ*_1_, given a particular value for the sigma-to-mean ratio *r* the Signal-Detection model of 4AFC defined by Eqs. (1) and (2). For example, when *r* = 0 and *δ* = 0, *μ*_*S*_ must equal 0.0393 for a first-response accuracy of 0.260. Given *r* = 0, *δ* = 0 and *μ*_*S*_ = 0.0393, the same Signal-Detection model predicts that second-response accuracy should be *Ψ*_2_ = 0.253.

I increased the value of *r* from 0 to 1.0, in steps of 0.1, and visual inspection revealed a roughly linear decline in the corresponding second-response accuracies. This procedure was repeated for first-response accuracies of 0.62, 0.84 and 0.96. For all of these first-response accuracies, second-response accuracy was found to decrease linearly with *r*. (NB: Precision limitations precluded use of *r* ⩾ 0.3 when *Ψ*_1_ = 0.96.)

The foregoing analysis was then automated, separately for each observer, so that the function mapping *r* to *Ψ*_2_ was fit with a regression line for first-choice accuracies 0.26, 0.27, … , 0.99 − *δ*, where *δ* was the frequency of finger errors. The largest values of *r* (up to a maximum of 1.0) compatible with our limited precision were used with each first-choice accuracy.

The gradients and intercepts of these regression lines were then plotted as functions of first-choice accuracy. These functions proved to be well fit by a fourth- and second-order polynomial, respectively. Thus, using the (eight) coefficients of these polynomials, we were able to compute SDT’s prediction for the function *Ψ*_2_(*Ψ*_1_;*r*), mapping first-response accuracy to second-response accuracy, given any arbitrary sigma-to-mean ratio *r*. For each observer, this function was used to find the value of *r* that maximized the likelihood of the observed second-response accuracies *P*_2_, given the observed first-response accuracies *P*_1_. Generalized log likelihood-ratios appear in Table D1.

## Footnotes

1 Targets were 0.01-s, 2.7-cd/m^2^ flashes superimposed on a 34-cd/m^2^ (i.e., 10 fL) background. Four observers performed the task from three different viewing distances, yielding 12 pairs of first- and second-response accuracy.

2 NB: I prefer the reciprocal to Green and Swetss’ (1966) terminology because it is not undefined for constant noise. That is, if *σ*_*X*_ = *σ*, ∀*X*, then *r* = 0.

3 For any *N* independent, identically distributed trials, the likelihood *l*_*i*_, of observing a frequency *P*_*i*_, of correct *i*th-responses is given by

(12) $$l_{i}=\left(\begin{array}{c} N\\ {\mathrm{NP}}_{i} \end{array}\right)\Psi_{i}^{{\mathrm{NP}}_{i}}{\left(1-\Psi_{i}\right)}^{N(1-P_{i})}\text{,}\;i=1\text{,}2\text{;}$$

where *Ψ*_*i*_ is the (true) probability of a correct *i*th-response. Note that *l*_1_ and *l*_2_ are not independent. The *joint* likelihood *l*, of observing frequencies *P*_1_ and *P*_2_ is given by

(13) $$l_{i}=\left(\begin{array}{c} N\\ {\mathrm{NP}}_{1}\text{,}{\mathrm{NP}}_{2}\text{,}N[1-P_{1}-P_{2}] \end{array}\right)\Psi_{1}^{{\mathrm{NP}}_{1}}\Psi_{2}^{{\mathrm{NP}}_{2}}{\left(1-\Psi_{1}-\Psi_{2}\right)}^{N(1-P_{1}-P_{2})}\text{.}$$

Finally, it should be noted that an upper bound for likelihood can be obtained by substituting *NP*_*i*_ for each *Ψ*_*i*_ in the preceding equations.
